# Death Quickly Produced without the Inhalation of Chloroform, in the First Stage of Natural Labor

**Published:** 1859-02

**Authors:** James H. Aveling

**Affiliations:** Honorary Member of the Obstetric Societies of Dublin and Edinburgh


*Death quickly produced without the Inhalation of Chloroform, in the First Stage of Natural Labor.* By JAMES H. AVELING, M. D., M. R. C. S. L., Honorary Member of the Obstetric Societies of Dublin and Edinburgh.

Having heard that an old patient of mine had died suddenly during labor at Charlton Brook, in the parish of Ecclesfield, where I was in practice three years ago, I this morning drove over and learnt the following particulars:

"My name is Mary K——: I was in the room at the death of Mrs. Ann G——. She sent for me about five o'clock in the evening, and told me that she had felt 'crotchety' all day. She was at her full time. I thought the pains sufficiently bad to advise her to send for her doctor. The surgeon came, and said it would not be over yet, and went away to see another patient. He returned between six and seven, and said she was still lingering. The pains seemed very severe, and Mrs. G. was very restless; sometimes she was on the bed, then on the chair, and then on her knees on the floor. She complained now and then of difficulty of breathing, and made a noise like 'croup.'—She also felt faint, and had some gruel and brandy. About two o'-clock in the morning the surgeon and husband were suddenly summoned up stairs, and the latter had only time to put his arm around Mrs. G., who was on the floor, to support her, when she 'shot out her legs,' and fell back gasping, and died instantly."

Had I been still living in Ecclesfield, I should have had this patient to attend; and as I am a great advocate for the administration of chloroform in labor, I should in all probability have given it in this case. Had I done so, and death had still occurred, chloroform would have been set down as the cause, and Dr. Lee would have had the opportunity of recording the history of a case in which death was quickly produced by the inhalation of chloroform, etc., in England. Neither in this nor in the case which took place in Scotland was any examination of the body made; and how without this it was found possible in the latter case to attribute with certainty the cause of death to chloroform, seems rather obscure.

It is not, however, for me here to enter upon what are or may have been the causes of sudden death in labor. My only wish is to place the case of Mrs. G. on record just now, because of its similarity to the one communicated by Dr. Lee, and also to allay in the minds of those practitioners who have taken fright at Dr. Lee's case, any fear they may have in giving chloroform for the future in labor.

I am so convinced from considerable experience, that women who have taken chloroform during parturition recover so very much better and faster than those who have not, that I should be very sorry to see its administration diminished in frequency, and its beneficial effects unnecessarily curtailed by a case in which the cause of death has been, without due examination, attributed to chloroform.

I should have been glad to have given a more professional account of the case of Mrs. G.; but the surgeon who attended her is just now on the continent, and I have no opportunity of getting information from him.

